# Grazing intensity drives plant diversity but does not affect forage production in a natural grassland dominated by the tussock-forming grass *Andropogon lateralis* Nees

**DOI:** 10.1038/s41598-021-96208-8

**Published:** 2021-08-18

**Authors:** Pablo Giliard Zanella, Luis Henrique Paim Della Giustina Junior, Cassiano Eduardo Pinto, Tiago Celso Baldissera, Simone Silmara Werner, Fabio Cervo Garagorry, Martín Jaurena, Fernando Alfredo Lattanzi, André Fischer Sbrissia

**Affiliations:** 1 Animal Science Department, Santa Catarina State University (UDESC/CAV), Av. Luiz de Camões, 2090, Lages, Santa Catarina 88520-000 Brazil; 2 Company of Agricultural Research and Rural Extension of Santa Catarina State (EPAGRI), Rua João José Godinho, sn – Morro do Posto, Lages, Santa Catarina 88502-970 Brazil; 3 Brazilian Agricultural Research Corporation (EMBRAPA), Rodovia BR-153, km 632,9, Caixa Postal 242, Bagé, Rio Grande Do Sul 96401-970 Brazil; 4 Instituto Nacional de Investigación Agropecuaria (INIA), Programa Pasturas y Forrajes, Estación Experimental INIA Tacuarembó, Tacuarembó, Uruguay; 5 Instituto Nacional de Investigación Agropecuaria (INIA), Programa Pasturas y Forrajes, Estación Experimental INIA La Estanzuela, Colonia, Uruguay

**Keywords:** Grassland ecology, Biodiversity, Agroecology

## Abstract

*Andropogon lateralis* is a tall and highly plastic tussock-forming grass native from southern South America. It is a frequent component of Campos and Subtropical highland grasslands that often becomes dominant under lax grazing regimes. The aim of this work was to analyze the response of species diversity and forage production of a natural grassland dominated by *A. lateralis* to a wide range of grazing intensity. We hypothesized that species diversity and forage production would both peak at the intermediate canopy heights determined by grazing regimes of moderate intensity. A grazing experiment was conducted in a highland grassland with mesothermal humid climate at 922 masl (Atlantic Forest biome, Santa Catarina state, Brazil) that comprised 87 species from 20 families but had 50% of its standing biomass accounted by *A. lateralis*. Four pre-/post-grazing canopy heights—12/7, 20/12, 28/17, and 36/22 cm (measured on *A. lateralis*)—were arranged in a complete randomized block design with four replications, and intermittently stocked with beef heifers from October 2015 to October 2017. *Andropogon lateralis* cover decreased (from 75 to 50%), and species richness increased (15–25 species m^−2^) as canopy height decreased. Grazing intensity did not affect annual forage production (4.2 Mg DM ha^−1^). This natural grassland dominated by *A. lateralis* had a high capacity to adjust to grazing regimes of contrasting intensity, maintaining forage production stable over a wide range of canopy heights. However, to prevent losses in floristic diversity, such grassland should not be grazed at canopy heights higher than 28 cm.

## Introduction

Humid natural grasslands, which have become rare worldwide, harbour a large diversity of fauna and flora. In southern Brazil, natural grasslands are the base of complex agroecosystems that support extensive livestock production and a range of other valuable environmental services in the Pampa and Atlantic Forest biomes. In the latter, the mesothermic humid Highland grasslands that extend at altitudes above 800 masl over some 1.374 million hectares^1,2^, characterized by estepe formation, are the habitat of 1161 species, of which 107 were endemic^2^, and 76 listed as endangered. Such unexpectedly high endemism was attributed to the large number of ecological niches in the tropical–temperate ecotone^3^.

In a study involving 40 locations on several continents^4^ it was found that grazing can exert substantial influence on biodiversity in agroecosystems, for instance, preventing species exclusion by reducing competition for light. At the same time, it is well known that grazing management, grazing intensity in particular, greatly influences not only the botanical composition but also the harvested yield of pastures^5–9^. For this reason, agroecosystems based on the use of highly diverse natural grasslands aim to design grazing management strategies that couple high enough animal production so that economic sustainability is ensured with the preservation of native species that provides the basis for resilience^10^.

*Andropogon lateralis* Nees is a tall tussock-forming perennial C4 grass native to South America. It is well accepted by grazing animals, presents an extremely high phenotypic plasticity showing large variations in specific leaf area (SLA) and leaf dry matter content (LDMC), and thus being able to display either a competing or a resource-conservation functional strategies depending on environmental conditions^11^. For instance, under lax grazing it conserves resources by reducing SLA and increasing LDMC, which along its tall stature confers such a competitive advantage that it often becomes highly dominant in large areas^12–14^.

The effects of grazing intensity on community structure have been found to mainly depend on, first, the ability of species to regrowth after a grazing event, and secondly, on the selectivity of the grazing animal for patches or species, both processes strongly interacting with forage biomass availability^15^. The often used humped-back model suggests that plant diversity in natural grassland ecosystems peaks at intermediate biomass levels^16^. Likewise, the intermediate disturbance hypothesis postulates that the highest diversity is maintained at intermediate scales of disturbance^17^. At community level, the replacement of few dominants by several subordinate species often occurs due to moderate grazing intensity and alleviating light limitation^4^.

In spite of its extension and relevance, few studies have focused on the response of natural grasslands dominated by *A. lateralis* to various grazing managements. In consequence, little is known about the processes determining species composition and forage production in these communities. Further, the particularly mild and productive climate in which these natural grasslands occur (> 1000 mm annual precipitation, no dry season, rare snowfall) difficults to extrapolate inferences from other studies. For instance, in such a humid climate, the intensity of competition for nutrients (nitrogen in particular) and for light, as well as the adaptive responses of different native species to nutrient deficiency and shading, could be much more important in determining competitive outcomes than in rangelands with typically semiarid and more continental climates (e.g. ^5–7,18–20^). Grazing intensity, with its strong effects on animal selectivity and plant growth^21^, and thus on (the spatial heterogeneity of both canopy height and nutrient (especially nitrogen) cycling, could therefore have an exacerbated role in modulating inter-specific relationships in these grasslands.

The aim of this work was to analyze the response of species diversity and herbage production of a natural grassland dominated by *A. lateralis* to a wide range of grazing intensities. We hypothesized that species diversity and forage production would both peak at the intermediate canopy heights determined by grazing regimes of moderate intensity, because under lax grazing intensity, species able to become tall and unpalatable, and conserve nutrients, would become dominant, whereas under too intense grazing only a few species with prostate habits would survive.

## Results

### Forage production

The annual production of forage was unaffected by grazing intensity and similar in both years (p > 0.05; Table 1), averaging 4236 kg DM ha^−1^.

**Table 1:** Forage production (kg DM ha^−1^ year^−1^) in a Highland grassland dominated by *Andropogon lateralis* Nees and managed under different canopy heights and intermittent stocking grazing in two growing seasons (GS).

Management height	12 cm	20 cm	28 cm	36 cm
1st GS (2015/16)	4124	3794	4177	3878
2nd GS (2016/17)	3644	5102	4017	5153

Mean = 4236 kg DM ha^−1^ year^−1^ and SEM = 1434 kg DM ha^−1^ year^−1^.

### Floristic composition

A total of 87 species distributed in 20 families were identified during the experiment. The largest number of species was in the Poaceae family (23 genera, 37 species), followed by the Asteraceae (16 genera, 19 species). The Fabaceae and Cyperaceae had five and four species in four and three genera, respectively. The Apiaceae and Convolvulaceae families were represented by three species, and Oxalidaceae and Rubiaceae by two species. All other families had only one species (Supplementary material).

On average, 50% of the pasture cover was *A. lateralis.* Ten species comprised over 90% of the forage mass (Table 2). The only non-grass was *Ulex europaeus*, a cosmopolitan invasive legume shrub native from Europe^22^. Two of the most important grasses were from the C3 physiological group pathway (*Anthoxanthum odoratum* and *Piptochaetium montevidense*), and the others were C4. The remaining species represented less than 1% of the total forage mass.

**Table 2:** Rank consistency index (Cr)^51^ and proportion of species (%) in the mean forage mass of the final two assessments (autumn and spring 2017) undertaken in a Highland grassland dominated by *Andropogon lateralis* Nees and managed under different canopy heights and intermittent stocking grazing.

Species	Cr	Canopy height management				Mean
		12 cm	20 cm	28 cm	36 cm	
*Andropogon lateralis*	0.95	[44.1]	[67.7]	[73.3]	[74.7]	65.0
*Paspalum notatum*	− 0.03	[16.3]	[5.3]	[4.0]	[1.7]	6.8
*Axonopus compressus*	− 0.68	[9.2]	[4.7]	[2.3]	[3.6]	5.0
Anthoxanthum odoratum*	− 0.77	[4.0]	[2.5]	[3.9]	[4.1]	3.6
*Axonopus affinis*	− 0.48	[7.5]	2.2	1.4	0.1	2.8
*Paspalum plicatulum*	− 0.88	3.9	1.6	1.9	1.3	2.2
*Piptochaetium montevidense*	− 0.94	1.3	2.0	[2.7]	0.9	1.7
*Erianthus angustifolius*	− 0.79	0.0	1.0	0.0	[5.7]	1.7
Ulex europaeus*	− 0.95	0.6	[3.1]	0.4	0.7	1.2
*Paspalum dilatatum*	− 0.98	0.5	1.8	1.0	0.6	1.0
Sum		87.4	91.9	90.9	93.4	** ~ 91%**
Dead material	0.53	2.5	14.7	17.9	20.8	14.0

The percentage of the most important species was calculated excluding the dead material component; however, as there was considerable participation of this in the forage mass, the percentage and Cr of this component based on the total forage mass were also calculated. *Exotic species. The five most important species at each management height are given in brackets.

The dead material component showed significant participation in the forage mass, especially at taller canopy heights, reaching just over 20% of the forage mass in the 36 cm canopy height.

### Species-area curves

**Figure 1:**
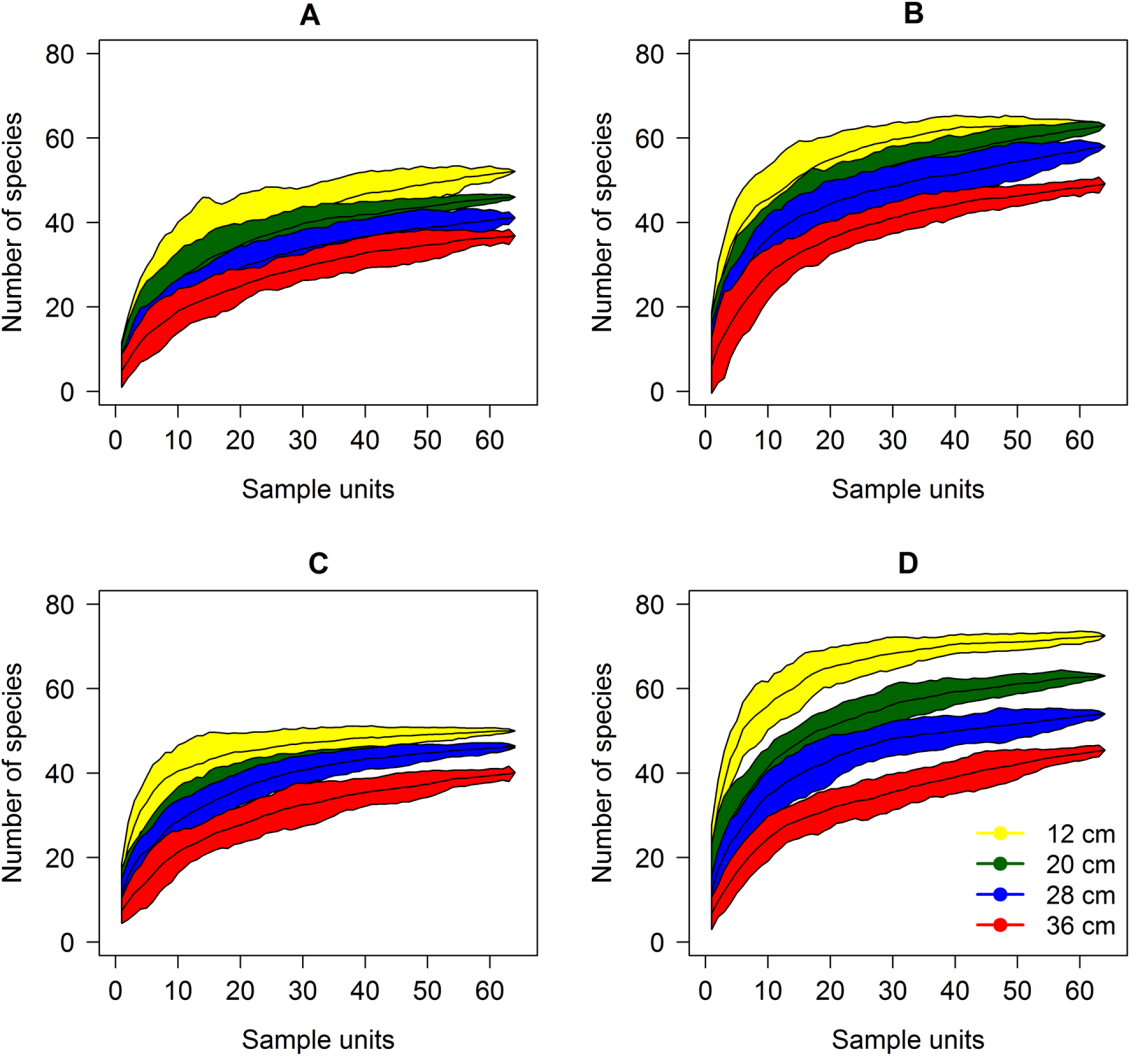
Species accumulation curves in a Highland grassland dominated by *Andropogon lateralis* Nees managed under different canopy heights. Evaluation seasons: autumn 2016 (**A**), spring 2016 (**B**), autumn 2017 (**C**), and spring 2017 (**D**). The number of species was considered distinct among canopy heights when confidence intervals of the species accumulation curve did not overlap. This figure was created by the authors using the R environment version 4.1.0 (https://www.R-project.org/) and the vegan package version 2.5-7 (https://CRAN.R-project.org/package=vegan).

Species accumulation curves differed among seasons and grazing intensity treatments (Fig. 1). There was a greater distance between accumulation curves in spring 2017, two years after the experiment started, with differences already apparent at the plot level (4 m^2^) and on the 15 m^2^ scale, yielding a difference of approximately 30 species. The number of species was also higher in spring than in autumn.

### Vegetation cover and tussock dynamics of *A. lateralis*

*A. lateralis* cover varied rapidly and importantly in response to grazing management, with some lesser variation due to season (interaction: *P* < 0.0001). Already 6 months after the beginning of the experiment, an increased *A. lateralis* cover was observed under the most lax grazing regime (pre-grazing height 36 cm), whereas cover was lowest in the most intensive grazing regime (12 cm) and intermediate in the other two treatments (Table 3). This trend was observed in all subsequent assessments, even though during the second year the cover of *A. lateralis* decreased somewhat in the more lax grazing regime.

**Table 3:** Percentage of vegetation cover by *Andropogon lateralis* Nees in a natural grassland managed under different canopy heights and intermittent stocking grazing.

Management height	Initial	1st year (2016)		2nd year (2017)		SEM*
		Autumn	Spring	Autumn	Spring	
12 cm	43.2	42.1 aC	39.9 aC	42.4 aD	43.6 aD	6.0
20 cm	52.5	66.9 aB	62.7 aB	56.4 bC	54.1 bC	5.6
28 cm	50.2	71.7 aB	66.3 abB	63.8 bB	64.5 bB	6.5
36 cm	46.8	87.1 aA	74.3 bA	74.6 bA	76.9 bA	5.8
SEM		7.5	6.0	7.0	5.7	

*SEM – standard error of the mean. Means followed by the same letters in lowercase (rows) and uppercase (columns) do not differ among themselves at 5% significance.

**Figure 2:**
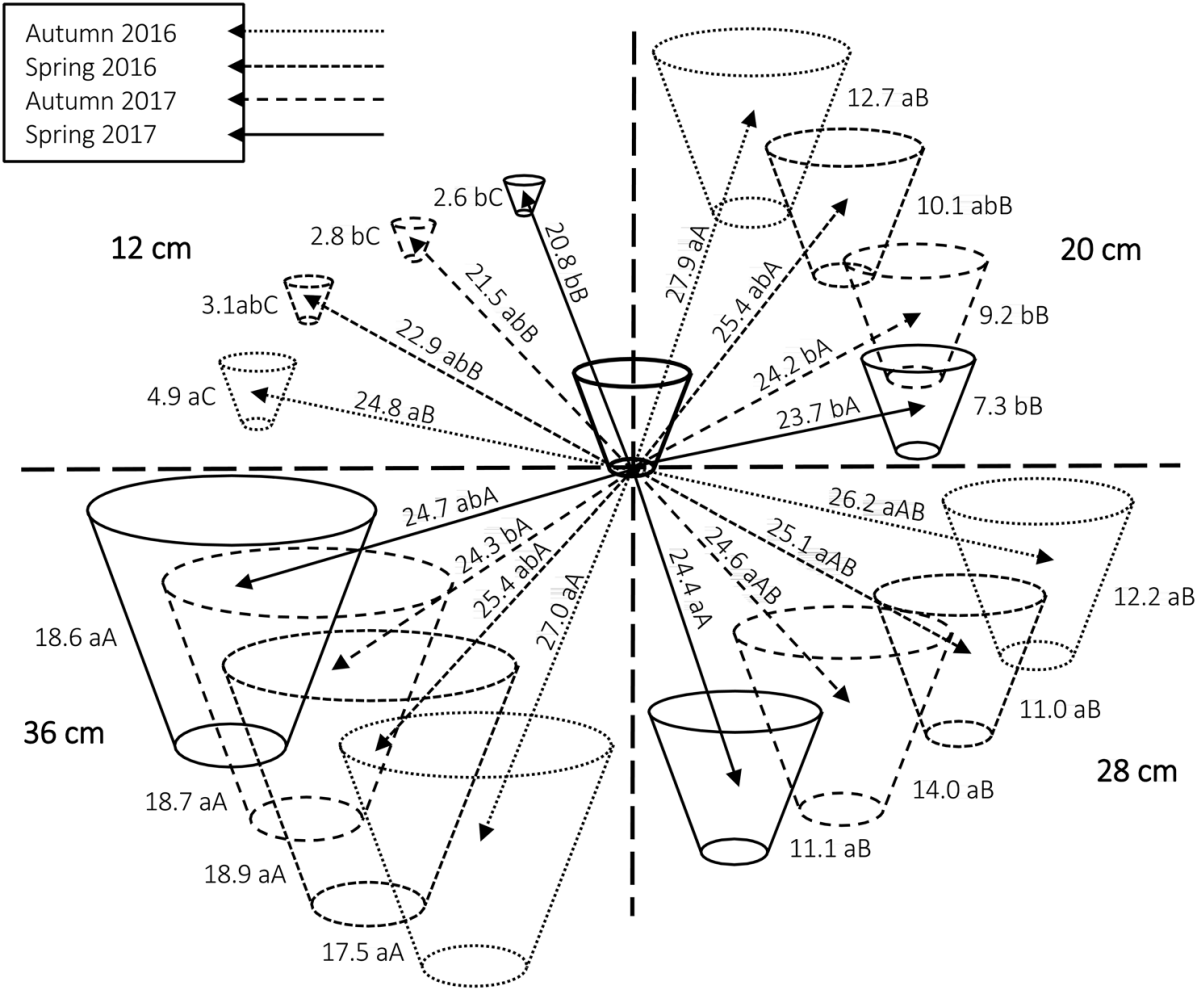
Changes in tussock volume (represented by cones; dm^3^) across seasons (each quadrant corresponds to a treatment canopy height) and inter-tussocks distance (represented by arrows; cm) of *Andropogon lateralis*. Values followed by the same letter do not differ. Lowercase letters compare seasons within canopy heights and uppercase letters compare canopy heights within seasons. The central cone represents the average tussock volumes before the implementation of treatments in August 2015. This figure was created by the authors using the Microsoft® Word for Mac, 16.50 (https://www.office.com/launch/word?auth=2).

Tussock volume showed similar, correlative changes than cover, becoming larger the lesser the grazing intensity with some minor variation between seasons (interaction: *P* = 0.0344; Fig. 2). Over time, tussock volume decreased in the two most intense grazing treatments (12 and 20 cm), with the lowest values observed in spring 2017. The mean distance between tussocks varied only a few centimeters between treatments and seasons, although the effect was statistically significant (*P* < 0.0001). The average distance between tussocks decreased over time for the 12 and 20 cm treatments.

### Indices for vegetation community

There was an interaction between grazing intensity and season in species richness (*P* = 0.0011). Grazing intensity and season had significant effects on species diversity (*P* < 0.0001 and *P* < 0.0001, respectively), dominance (*P* < 0.0001 and *P* = 0.0066, respectively), and heterogeneity (*P* < 0.0001 and *P* = 0.0120, respectively) (Table 4).

**Table 4:** Richness (number of species m^−2^), diversity (Shannon index, H′), dominance (1-Simpson index × 100), and structural heterogeneity (coefficient of variance [CV%] of height) per square meter (1 m^2^) of a Highland grassland dominated by *Andropogon lateralis* Nees managed under different canopy heights and intermittent stocking grazing during two years of evaluation.

Management height	Initial	1st year (2016)		2nd year (2017)		Mean	SEM*
		Autumn	Spring	Autumn	Spring		
**Richness (number of species m**^−**2**^**)**							
12 cm	18	17 cA	22 bA	20 bA	25 aA	21	2.8
20 cm	19	16 bAB	20 aA	19 abA	22 aAB	19	3.1
28 cm	18	15 bAB	20 aA	18 aA	20 aB	18	2.5
36 cm	18	14 aB	16 aB	15 aB	15 aC	15	3.2
Mean		16	19	18	20		
SEM		2.5	3.3	2.7	2.9		
**Diversity (Shannon index, H′)**							
12 cm	1.45	1.43	1.62	1.61	1.72	1.59 A	0.24
20 cm	1.48	1.27	1.44	1.49	1.45	1.41 B	0.18
28 cm	1.44	1.20	1.39	1.33	1.38	1.32 B	0.17
36 cm	1.34	1.09	1.26	1.13	1.18	1.17 C	0.17
Mean		1.25 b	1.43 a	1.39 a	1.43 a		
SEM		0.27	0.22	0.22	0.24		
Dominance (1 − Simpson*100)							
12 cm	37.9	33.7	31.8	31.8	30.9	32.7 C	7.7
20 cm	39.6	44.6	39.6	36.8	41.1	40.5 B	6.9
28 cm	39.4	47.2	41.6	42.0	42.2	43.2 AB	7.7
36 cm	43.3	49.7	42.4	47.2	45.4	46.2 A	7.6
Mean		44.4 a	38.8 b	39.5 b	39.9 b		
SEM		9.8	8.0	8.0	9.3		
**Heterogeneity (CV% of canopy height)**							
12 cm	31.0	36.0	36.9	43.6	33.8	37.3 A	6.5
20 cm	24.9	35.1	32.2	41.5	33.4	35.8 A	10.1
28 cm	27.1	33.5	28.0	27.4	27.2	29.8 B	7.9
36 cm	23.1	26.3	26.8	28.5	25.2	27.2 B	5.3
Mean		32.7 ab	31.0 b	36.1 a	30.3 b		
SEM		10.2	8.8	8.1	7.5		

*SEM – standard error of the mean. Means followed by the same letters in lowercase (rows) and uppercase (columns) do not differ among themselves at 5% significance.

### Species richness and structural heterogeneity

**Figure 3:**
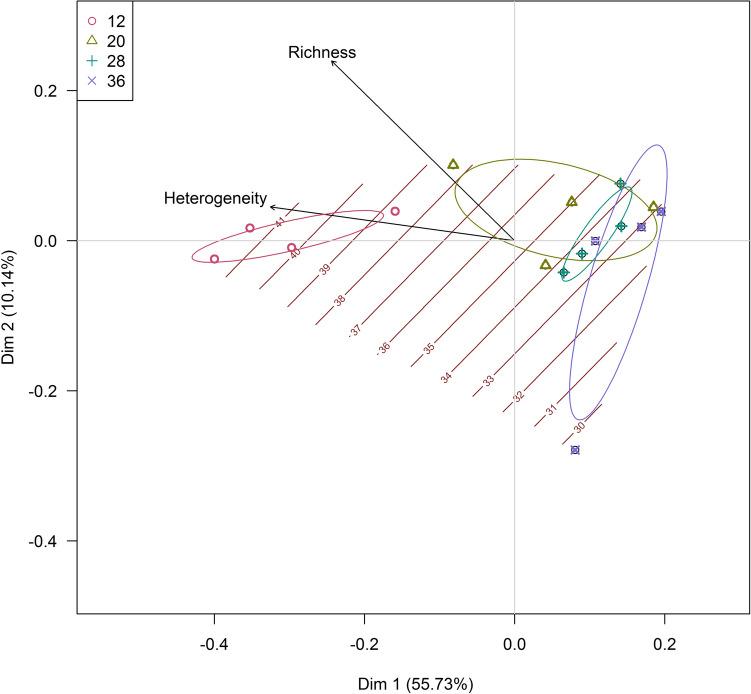
Principal coordinate analysis (PCoA) with ordination for species richness (Richness) and structural heterogeneity (Heterogeneity) at the plot level of 4 m^2^, in a Highland grassland dominated by *Andropogon lateralis* Nees managed at different canopy heights. The grading levels relate to the number of species and the ellipses to the treatments. The Bray–Curtis method was used, with a restricted maximum likelihood value for species richness of 44.63. This figure was created by the authors using the R environment version 4.1.0 (https://www.R-project.org/), the vegan version 2.5-7 (https://CRAN.R-project.org/package=vegan) and BiodiversityR version 2.13-1 (https://cran.r-project.org/web/packages/BiodiversityR/index.html) packages.

Canopy height of 12 cm was positively associated with both structural heterogeneity and species richness. Despite being more heterogeneous (Table 4), the 20 cm canopy height was associated with the 28 and 36 cm treatments, as shown by the overlapping ellipses (Fig. 3).

Species richness was highest in the most intense grazing treatment (12 cm) and lowest in the most lax (36 cm), with intermediate values in the intermediate grazing intensities (20 and 28 cm). The difference between grazing treatments was maximal at the end of the experiment: 25 and 15 species, respectively for the most intense and lax grazing treatments (Table 4). Species diversity showed a similar behavior, with highest values at 12 cm, lowest at 36 cm, and intermediate at 20 and 28 cm.

## Discussion

During both experimental years, no difference was observed in forage production across grazing intensity treatments (Table 1). Hence, forage production was buffered over a wide range of canopy heights. This response has already been reported for monospecific grasslands^8,21,23^ and associated with a trade-off between gross growth and senescence fluxes results in a relatively stable net forage production (= harvested or consumed herbage), and by underlying tiller size/density compensation^24^. Although our study produced a large variation in canopy structure, reflected in heights ranging from 12/7 to 36/22 cm, *A. lateralis* remained dominant even at the highest grazing intensity (Table 2), confirming the high plasticity reported for this species^11^.

In spite of a stable forage production, grazing intensity did promote strong changes in the plant community. At the lowest grazing intensity (36 cm canopy height), there was almost no temporal or spatial variation in *A. lateralis* cover (maximal), nor in richness and diversity (minimal), over the two years of the study (Tables 3 and 4). But as grazing intensity increased, and canopy height became shorter, it increased the participation and number of other species, especially in the lower stratum (Table 3). This effect was evident in both autumn and spring, becoming more marked as the experiment progressed. Maximal richness and diversity were thus observed in the shortest canopies at the end of the experimental period. Since *A. lateralis*, like other tall tufted grass, is highly competitive for light^11,25^ under lax grazing regime, this response probably occurred as a result of changes in the light environment brought about by the reduction in *A. lateralis* cover that favored the development of subordinate, more shaded species^26,27^. However, concomitant effects of a possible higher nutrient availability due to more animal excreta at higher grazing intensities can not be excluded. Both height and shoot nitrogen concentration are strong determinants of individual and species dominance in diverse communities^28,29^.

Under high grazing intensity, the mean distance between *A. lateralis* tussocks became shorter (Fig. 2). This may reflect central tussock dieback, a phenomenon that results from the death of the tussock centre and its subsequent fragmentation^30^. Therefore, a higher light availability penetrating into the canopy and increasing the participation of species already present in the forage mass or the appearance of new species would have resulted not only from lower the tussock height but also from their smaller basal cover. Increases in the number of species in short canopies has been reported^27^, demonstrating that grazing can be used as a practical tool to maintain or even increase plant diversity.

Grasses are more competitive than other herbaceous species^5^; thus, species richness can be affected by grass dominance level, especially tussock-forming species, which have canopy advantages in terms of light competition^7,31,32^. The greater the abundance of dominant species, the more severe the interspecific competition^33,34^ but grazing can affect species distribution patterns in the canopy, influencing competition processes^35^. Therefore, it is important to understand the mechanisms that drive grazing-generated changes (Fig. 3) based on species composition characteristics^27,36^.

The humped-back model indicates that plant diversity is at its maximum at an intermediate biomass level^16^. Based on this, we expected to observe a succession of structures across the grazing intensity treatments, from shorter and homogeneous at high intensity, to bimodal and heterogeneous at moderate intensities, to taller and homogeneous at low intensities. However, structural heterogeneity was highest in the shorter sward (Fig. 3), as well as species richness and diversity because of the reduced dominance of *A. lateralis* and increased participation of prostate species. This supports the idea that as long as grazing intensity creates spatial heterogeneity, species diversity can increase. The greater grazing intensity used in our study was not sufficient to reduce heterogeneity, species richness or diversity.

## Conclusions

The forage production of a natural grassland dominated by A. *lateralis* in the Highlands of the Atlantic Forest proved to be remarkably stable over two years across a range of grazing intensities. Conversely, botanical composition varied markedly, with lesser richness and diversity, and higher cover of *A. lateralis,* as the grazing intensity became more lax and thus the canopy taller. Maximal diversity, as well as structural heterogeneity, were found at the highest grazing intensity and thus shorter canopy (12 cm treatment). *A. lateralis* tussocks were smaller and somewhat closer apart in this treatment, representing less than half of the forage mass. Under lax grazing, *A. lateralis* tussocks were large, and had accounted for over 75% of the standing biomass. This evidences the great capacity of these natural grasslands to adapt their structure over a great range of grazing intensities by changing both its species composition and functionality of the dominant species. Thus, grazing regime can be managed to increase the diversity of plant species while maintaining forage production of these natural grasslands by keeping canopy heights below 28 cm.

## Material and methods

This manuscript reports experiments with plants and animals and complies with national and institutional guidelines. All procedures involving animals in this study were approved by the Animal Ethics Committee of the Santa Catarina State University (6,241,030,918). We state also that all plant species used in this experiment is native and endemic in the region of study.

### Area, experimental design, and management

**Figure 4:**
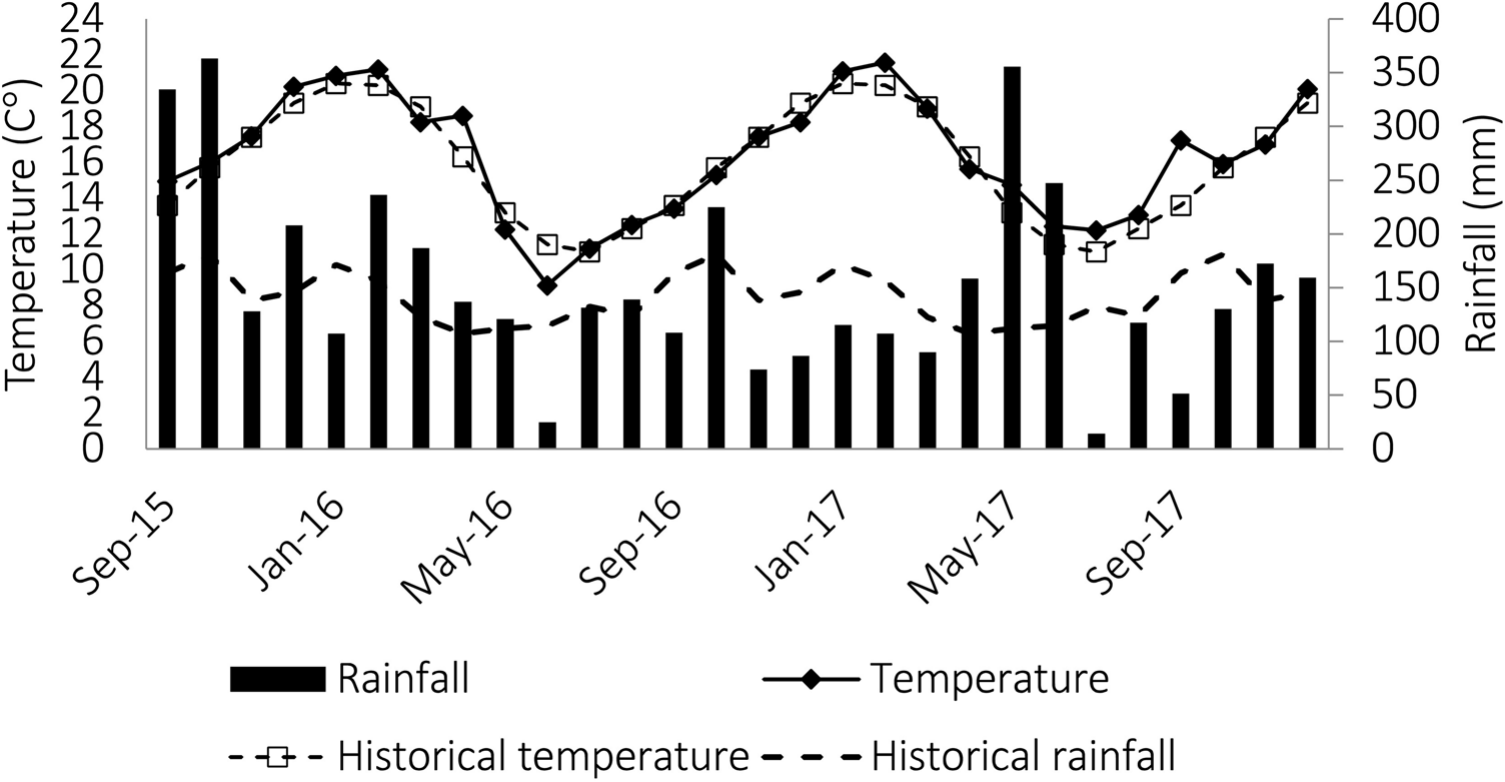
Temperature and rainfall data for the study site relating to the experimental period, and historical climate data from the previous 58 years. Climate data was obtained from the weather station of the Santa Catarina Agricultural Research and Rural Extension Company (Epagri/EEL).

The experiment was carried out at Company of Agricultural Research and Rural Extension of Santa Catarina (Epagri/EEL), located in Lages (Santa Catarina State, Brazil; 27° 47′ 55′′ S, 50° 19′ 25′′ W; altitude, 922 m; and annual rainfall, 1,668 mm). The region’s climate is humid mesothermal (Cfb; Köppen classification), with cool winters, mild summers, and well-distributed rainfall throughout the year^37^. The experimental area was homogeneous in relation to the type of soil, with a slope of 7.5% + − 2.3, and the soil is classified as Humudept (with an umbric epipedon) (Soil Taxonomy). Soil fertility parameters were measured using samples taken from the upper 0–10 cm layer and were as follows: pH (SMP) = 4.8, P = 3.4 mg L^−1^, K = 114 mg L^−1^, organic matter (OM) = 5%, Al = 3.1 cmol_c_ L^−1^, Ca = 4.3 cmol_c_ L^−1^, and Mg = 2.1 cmol_c_ L^−1^. Historical climatic data and data from the experimental period were obtained from a weather station located 300 m from the study area (Fig. 4).

The experimental area was also homogeneous in relation to vegetation cover, which consisted of natural grassland predominated by *A. lateralis*^38^, with no history of anthropogenic use (ploughing, soil fertilization, fire, or species introductions) in the previous 40 years. Prior to the study, the experimental site was grazed by beef cattle, with no defined canopy height management target. The 14,000 m^2^ experimental area was divided into 16 paddocks, each 875 m^2^.

First grazing was carried out on 19 August 2015 for homogenization; the animals remained in the paddocks for three days and reduced the canopy height to 11.7 cm. This date was set as the start of the experimental period and lasted until October 2017. Flanders Red (La Rouge Flamande) dry cows were used for grazing, each weighing approximately 613 ± 92 kg.

The experiment was conducted in a randomized complete block design with four treatments and four replications. To test our hypothesis four pre-grazing canopy heights were chosen (12, 20, 28, and 36 cm), based on measuring only the predominant species (*A. lateralis*). Such canopy heights were defined to create contrasting grazing intensities where taller canopy height (36 cm) corresponded to a less intense or a lax grazing regime and the lower one (12 cm) a very intense or hard grazing regime. The intermittent stocking method was used^39^, with irregular rest periods (determined by canopy height). The criterion for grazing interruption was defined as a reduction in the pre-grazing canopy height by 40% (7.2, 12, 16.8, and 21.6 cm). This management target was set to allow grazing animals to access almost exclusively leaves, since approximately 90% of all stem components are concentrated in the lower half-stratum of the canopy^40,41^.

### Canopy height

Canopy height was monitored weekly using a sward stick^42^. These measurements were performed systematically along four evaluation lines (40 points per plot) of *A. lateralis* tussocks. Measurements of the lower stratum (inter-tussock gaps) followed the same methodology, but with 20 points per plot. When the average height in each plot reached the targets for each treatment canopy height (based on readings of *A. lateralis* only), the animals were placed in the plots to start grazing. After removing the animals from the plots, the post-grazing heights of *A. lateralis* and the inter-tussock stratum were recorded, following the same evaluation methodology.

### Forage production

Forage production was estimated by means of double sampling^43^ by the difference of pre and post-grazing herbage mass. Herbage mass was estimated from the cutting of four paired samples per plot and visual estimate of 20 samples obtained systematically in four lines of evaluation per plot, in pre and post-grazing conditions, in all grazing cycles. The cuts were made close to the ground, in frames of 0.25 m^2^ (0.5 × 0.5 m). After, the samples were dried in an oven with forced air ventilation at 55 °C for 72 h to determine the dry matter content. To calibrate the visual estimate, regression equations of the estimated forage mass with the forage mass obtained in the cuts (kg DM ha^−1^) were constructed. The forage production (FP) was calculated by difference from the estimated calibrated forage mass, discounting the pre-grazing (FM_PRE_) forage mass from the post-grazing (FM_POST_) forage mass of the previous cycle (FP = FM_PRE_ cycle^n ^− FM_POST_ cycle^n−1^). To obtain the forage production of the year, the production of all cycles of the growing season plus the winter production were added, starting from the post-grazing of the 1st cycle until the pre-grazing of the 1st cycle of the subsequent year, totaling two production years for the 2015/16 and 2016/17 growing seasons.

### Vegetation cover and tussock dynamics of *A. lateralis*

The percentages of *A. lateralis* vegetation cover and tussock dynamics were evaluated in pre-grazing conditions at the beginning (October to November) and end (April to May) of the growing season (spring and autumn, respectively) for two consecutive years. The first of these, in October–November 2015, was characterized as the initial evaluation because no treatment effect had yet been observed. To assess the percentage of *A. lateralis* vegetation cover, four transects of 25 m (total 100 m) per plot were established, along which tussock size and gaps (the spaces between tussocks or tussock groups) were measured^44^. Twelve tussocks from the four transects were marked so that changes in base circumference, height, and crown projection diameter could be monitored, enabling the tussock volume to be calculated according to the following equation^45^:

$${\text{V}} = 1/3\Pi .{\text{h}}\left( {{\text{r}}^{2} + {\text{rR}} + {\text{R}}^{2} } \right)$$

where V is volume, h is distance from soil to the top of tussock, r is radius of basal area occupied by tussock, and R is radius of area at top of tussock canopy.

The average distance between tussocks was also evaluated according to the Point-Centred Quarter Method^46^, by measuring the distances from the four nearest tussocks to the reference tussock, one in each quadrant, thus allowing *A. lateralis* space occupancy to be more accurately estimated.

### Floristic composition

A floristic composition survey was carried out for two consecutive years, at the beginning and end of the growing seasons under pre-grazing conditions, to facilitate the identification of spring and autumn flowering species (winter and summer species, respectively). This procedure was adopted since the type of grassland studied here is characterized by a rare association of C3 and C4 species coexisting simultaneously^2^. Four squares of 0.25 m^2^ (0.5 × 0.5 m) were established at the centre of each plot and same transects as used in the tussock evaluations, totalling 4 m^2^ and 16 samples per plot (sampling unit). Floristic surveys were repeated in the same sampling units for each evaluation to verify the vegetation patterns and dynamics of the grassland community, using the Botanical analysis method (Botanal)^47^; this method represents the relationship between the composition of species in the area and their participation in the forage mass, expressed on a dry weight basis. Each sample was ranked according to participation of the most frequent species in the forage mass. Then, other species present within the sample squares were identified and attributed a notional percentage (1%), since they made no significant contribution to forage mass. Forage mass in the Botanal samples was estimated visually by two trained evaluators and then calibrated through a regression equation using forage mass samples (kg DM ha^−1^ estimated per kg DM^−1^ obtained from cutting samples) from locations adjacent to the sampling plots after each evaluation period^48^. Five pasture height readings were recorded per sample using a sward stick^42^.

### Vegetation community and heterogeneity indices

Plant community indices were calculated using the vegan^49^ and BiodiversityR^50^ packages in R statistical software^51^. The species accumulation curve was generated using the Specaccum function of the Vegan package and normalized by considering the number of species identified in the first evaluation. The rank consistency index (Cr) refers to the relative variation of species between treatments in the final two surveys^52^. Richness was estimated by counting the number of species, and it was estimated diversity (Shannon index, H′), and dominance (1-Simpson × 100 index) based on the proportion of species in the forage mass. Structural heterogeneity was calculated based on the pasture height of Botanal samples, which was expressed as a percentage variation of the mean height (coefficient of variation for canopy height).

### Statistical data analysis

Data analysis was performed using R environment^51^. The variables were adjusted by linear mixed models using the lme4 package^53^, considering block as a random effect, the canopy height, grazin condition (pré and post-grazing) and season or year of evaluation as fixed effects and different variance and covariance structures. The first measurement was used as a covariable for *A. lateralis* vegetation cover and tussock dynamics as well as for vegetation indices. The model selection was based on Akaike Information Criterion (AIC). Principal coordinate analysis (PCoA) was performed with the vegdist function of the vegan^49^ package using the Bray–Curtis method, the classification of species richness and structural heterogeneity at plot level (4 m^2^) were fitted and ellipses for the treatments were added.

## Data Availability

The datasets generated during this study are available from figshare^54^ https://doi.org/10.6084/m9.figshare.14055419.v1.

## References

[1] IBGE. Instituto Brasileiro de Geografia e Estatística - Censo Agro 2017. *IBGE | Censo Agro 2017, Dados preliminares* https://censos.ibge.gov.br/agro/2017/ (2017).

[2] Boldrini, I. I. *et al.* Flora. In *Biodiversidade dos Campos do Planalto das Araucárias* 39–94 (2009).

[3] Iganci JRV, Heiden G, Miotto STS, Pennington RT (2011). Campos de Cima da Serra: The Brazilian subtropical highland Grasslands show an unexpected level of plant endemism. Bot. J. Linn. Soc..

[4] Borer ET (2014). Herbivores and nutrients control grassland plant diversity via light limitation. Nature.

[5] Alhamad MN, Alrababah MA (2008). Defoliation and competition effects in a productivity gradient for a semiarid Mediterranean annual grassland community. Basic Appl. Ecol..

[6] Fedrigo JK (2017). Temporary grazing exclusion promotes rapid recovery of species richness and productivity in a long-term overgrazed Campos grassland. Restor. Ecol..

[7] Mavromihalis JA, Dorrough J, Clark SG, Turner V, Moxham C (2013). Manipulating livestock grazing to enhance native plant diversity and cover in native grasslands. Rangel. J..

[8] Bircham JS, Hodgson J (1983). The influence of sward condition on rates of herbage growth and senescence in mixed swards under continuous stocking management. Grass Forage Sci..

[9] Sbrissia AF, Duchini PG, Zanini GD, Santos GT, Padilha DA, Schmitt D (2018). Defoliation strategies in pastures submitted to intermittent stocking method: Underlying mechanisms buffering forage accumulation over a range of grazing heights. Crop Sci..

[10] Jaurena M (2021). Native grasslands at the core: A new paradigm of intensification for the Campos of Southern South America to increase economic and environmental sustainability. Front. Sustain. Food Syst..

[11] Cruz P (2010). Leaf traits as functional descriptors of the intensity of continuous grazing in native grasslands in the South of Brazil. Rangel. Ecol. Manag..

[12] Benitez, C. A. & Fernandez, J. G. *Espécies forrageiras de la pradera natural: Fenologia y respuesta a la frequência e severidad de corte* (1970).

[13] Herve, A. M. B. & Valls, J. F. M. *Genêro Andropogon L. (Gramineae) no Rio Grande do Sul*. *Anuario tecnico do Instituto de Pesquisas Zootecnicas Francisco Osorio* (1980).

[14] Zanin A, Longhi-Wagner HM (2011). Revisão de Andropogon (Poaceae - Andropogoneae) para o Brasil. Rodriguesia.

[15] Augustine DJ, McNaughton SJ (1998). Ungulate effects on the functional species composition of plant communities: Herbivore selectivity and plant tolerance. J. Wildl. Manag..

[16] Fraser LH (2015). Worldwide evidence of a unimodal relationship between productivity and plant species richness. Science.

[17] Connell JH (1978). Diversity in tropical rain forests and coral reefs: High diversity of trees and corals is maintained only in a nonequilibrium state. Science.

[18] Milchunas DG, Sala OE, Lauenroth WK (1988). A generalized model of the effects of grazing by large herbivores on grassland community structure. Am. Nat..

[19] Liu J (2015). Impacts of grazing by different large herbivores in grassland depend on plant species diversity. J. Appl. Ecol..

[20] Ren H, Schönbach P, Wan H, Gierus M, Taube F (2012). Effects of grazing intensity and environmental factors on species composition and diversity in typical Steppe of Inner Mongolia, China. PLoS ONE.

[21] Sbrissia AF, Silva SC, Schmitt D, Duchini PG (2020). Unravelling the relationship between a seasonal environment and the dynamics of forage growth in grazed swards. J. Agron. Crop Sci..

[22] Hernández-Lambraño RE, González-Moreno P, Sánchez-Agudo JÁ (2017). Towards the top: Niche expansion of *Taraxacum officinale* and *Ulex europaeus* in mountain regions of South America. Austral. Ecol..

[23] Pinto LFM (2001). Dinâmica do acúmulo de matéria seca em pastagens de Tifton 85 sob pastejo. Sci. Agric..

[24] Duchini PG, Guzatti GC, Ribeiro Filho HMN, Sbrissia AF (2014). Tiller size/density compensation in temperate climate grasses grown in monoculture or in intercropping systems under intermittent grazing. Grass Forage Sci..

[25] Briske DD, Anderson VJ (1992). Competitive ability of the bunchgrass *Schizachyrium scoparium* as affected by grazing history and defoliation. Vegetatio.

[26] Altesor A, Oesterheld M, Leoni E, Lezama F, Rodriguez C (2005). Effect of grazing on community structure and productivity of a Uruguayan grassland. Plant Ecol..

[27] Lezama F (2014). Variation of grazing-induced vegetation changes across a large-scale productivity gradient. J. Veg. Sci..

[28] Lattanzi FA (2012). 13C-labeling shows the effect of hierarchy on the carbon gain of individuals and functional groups in dense field stands. Ecology.

[29] Roscher C, Karlowsky S, Milcu A, Gessler A, Bachmann D, Jesch A (2019). Functional composition has stronger impact than species richness on carbon gain and allocation in experimental grasslands. PLoS ONE.

[30] Wan C, Sosebee RE (2000). Central dieback of the dryland bunchgrass *Eragrostis curvula* (weeping lovegrass) re-examined: The experimental clearance of tussock centres. J. Arid Environ..

[31] Angassa A (2014). Effects of grazing intensity and bush encroachment on herbaceous species and rangeland condition in Southern Ethiopia. L. Degrad. Dev..

[32] Schultz NL, Morgan JW, Lunt ID (2011). Effects of grazing exclusion on plant species richness and phytomass accumulation vary across a regional productivity gradient. J. Veg. Sci..

[33] Chaneton EJ, Facelli JM (1991). Disturbance effects on plant community diversity: Spatial scales and dominance hierarchies. Vegetatio.

[34] Tow, P. G. & Lazenby, A. *Competition and Succession in Pastures* (CAB International, 2001). doi:10.1079/9780851994413.0000.

[35] Briske DD, Hendrickson JR (1998). Does selective defoliation mediate competitive interactions in a semiarid savannah? A demographic evaluation. J. Veg. Sci..

[36] Baer SG, Blair JM, Collins SL (2016). Environmental heterogeneity has a weak effect on diversity during community assembly in tallgrass prairie. Ecol. Monogr..

[37] Alvares CA, Stape JL, Sentelhas PC, Gonçalves JLM, Sparovek G (2013). Köppen’s climate classification map for Brazil. Meteorol. Zeitschrift.

[38] Pallarés, O. R., Berretta, E. J. & Maraschin, G. The South American Campos ecosystem BT—Grasslands of the World. Grasslands of the World 1–49 (2005).

[39] Allen VG (2011). An international terminology for grazing lands and grazing animals. Grass Forage Sci..

[40] Zanini GD, Santos GT, Schmitt D, Padilha DA (2012). Distribuição de colmo na estrutura vertical de pastos de capim Aruana e azevém anual submetidos a pastejo intermitente por ovinos. Ciênc. Rural.

[41] Carvalho PCF (2013). Harry Stobbs Memorial Lecture: Can grazing behaviour support innovations in grassland management?. Trop. Grassl. Forrajes Trop..

[42] Barthram, G. T. Experimental techniques: The HFRO sward stick. In The Hill Farming Research Organization Biennial Report 1984/1985 29–30 (HFRO, 1985).

[43] Haydock KP, Shaw NH (1975). The comparative yield method for estimating dry matter yield of pasture. Aust. J. Exp. Agric..

[44] Williams RJ (1992). Gap dynamics in subalpine heathland and grassland vegetation in south-eastern *Australia*. J. Ecol..

[45] Derner JD, Briske DD, Polley HW (2012). Tiller organization within the tussock grass *Schizachyrium scoparium*: A field assessment of competition–cooperation tradeoffs. Botany.

[46] Mueller-Dombois, D. & Ellenberg, D. Aims and methods of vegetation ecology. In *Community Sampling: The Relevé Method* 45–66 (1974).

[47] Tothill JC, Hargreaves JNG, Jones RM, McDonald CK (1992). Botanal—A comprehensive sampling and computing procedure for estimating pasture yield and composition. 1. Field sampling. Trop. Agron. Tech. Mem..

[48] ’t Mannetje, L. Measuring biomass of grassland vegetation. In *Field and Laboratory Methods for Grassland and Animal Production Research* 151–177 (CABI, 2000). doi:10.1079/9780851993515.0151.

[49] Oksanen, F.J., Blanchet, G., Friendly, M., Kindt, R., Legendre, P. et al. vegan: Community Ecology Package. R package version 2.5-7. (2020). https://CRAN.R-project.org/package=vegan.

[50] Kindt, R. & Coe, R. Tree diversity analysis. A manual and software for common statistical methods for ecological and biodiversity studies. World Agroforestry Centre (ICRAF), Nairobi. ISBN: 92-9059-179-X (2005).

[51] R Core Team. R: A language and environment for statistical computing. R Foundation for Statistical Computing, Vienna, Austria. (2021). https://www.R-project.org/.

[52] Watkins AJ, Wilson JB (1994). Plant community structure, and its relation to the vertical complexity of communities: dominance/diversity and spatial rank consistency. Oikos.

[53] Bates D, Mächler M, Zurich E, Bolker BM, Walker SC (2015). Fitting linear mixed-effects models using lme4. J. Stat. Softw..

[54] Sbrissia, A. F., Zanella, P. G., Pinto, C. E., Baldissera, T. C. & Garagorry, F. C. Natural grasslands experiment - 2015 - 2017 - Pablo. figshare. 10.6084/m9.figshare.14055419.v1 (2021).

